# Structural features, kinetics and SAR study of radical scavenging and antioxidant activities of phenolic and anilinic compounds

**DOI:** 10.1186/1752-153X-7-53

**Published:** 2013-03-16

**Authors:** Hussein M Ali, Ahmed Abo-Shady, Hany A Sharaf Eldeen, Hany A Soror, Wafaa G Shousha, Osama A Abdel-Barry, Ahmed M Saleh

**Affiliations:** 1 Department of Agricultural Biochemistry, Faculty of Agriculture, Ain Shams University, Cairo, Egypt; 2 Faculty of Science, Helwan University, Cairo, Egypt; 3 Present address: Faculty of Science For Girls, Chemistry Department, Dammam University, Dammam, SA, 31113, P.O. Box 838, Saudi Arabia

**Keywords:** Antioxidant activity, Phenols, Anilines, DPPH, Hydroxyl radicals, Phenoxyl radicals, Structural features, SAR

## Abstract

**Background:**

Phenolic compounds are widely distributed in plant kingdom and constitute one of the most important classes of natural and synthetic antioxidants. In the present study fifty one natural and synthetic structurally variant phenolic, enolic and anilinic compounds were examined as antioxidants and radical scavengers against DPPH, hydroxyl and peroxyl radicals. The structural diversity of the used phenolic compounds includes monophenols with substituents frequently present in natural phenols e.g. alkyl, alkoxy, ester and carboxyl groups, besides many other electron donating and withdrawing groups, in addition to polyphenols with 1–3 hydroxyl groups and aminophenols. Some common groups e.g. alkyl, carboxyl, amino and second OH groups were incorporated in *ortho*, *meta* and *para* positions.

**Results:**

SAR study indicates that the most important structural feature of phenolic compounds required to possess good antiradical and antioxidant activities is the presence of a second hydroxyl or an amino group in *o*- or *p*-position because of their strong electron donating effect in these positions and the formation of a stable quinone-like products upon two hydrogen-atom transfer process; otherwise, the presence of a number of alkoxy (in *o* or *p*-position) and /or alkyl groups (in *o*, *m* or *p*-position) should be present to stabilize the resulted phenoxyl radical and reach good activity. Anilines showed also similar structural feature requirements as phenols to achieve good activities, except *o*-diamines which gave low activity because of the high energy of the resulted 1,2-dimine product upon the 2H-transfer process. Enols with ene-1,2-diol structure undergo the same process and give good activity. Good correlations were obtained between DPPH inhibition and inhibition of both OH and peroxyl radicals. In addition, good correlations were obtained between DPPH inhibition and antioxidant activities in sunflower oil and liver homogenate systems.

**Conclusions:**

In conclusion, the structures of good anti radical and antioxidant phenols and anilines are defined. The obtained good correlations imply that measuring anti DPPH activity can be used as a simple predictive test for the anti hydroxyl and peroxyl radical, and antioxidant activities. Kinetic measurements showed that strong antioxidants with high activity have also high reaction rates indicating that factors stabilizing the phenoxyl radicals lower also the activation energy of the hydrogen transfer process.

## Background

Phenolic compounds are widely distributed in plant kingdom and constitute one of the most important classes of natural (e.g. α-tocopherol, gallic acid and syringic acid) and synthetic (e.g. BHT, BHA and TBHQ) antioxidants [1-4]. Even simple phenolsshow wide variations in their chemical structures that include monophenols (e.g. cresols and eugenol), polyphenols (e.g. catechols and hydroquinones), substituted benzoic acid (e.g. salicylic and vanillic acids) and cinnamic acid (e.g. caffeic acid), and terpenoids (e.g. thymol and carvacrol). A few reports indicated that anilines also express radical scavenging and antioxidant activities [5-8]; where some of them present naturally in living cells (e.g. *o*- and *p*-aminobenzoic acids). Phenolic antioxidants are generally believed to form phenoxyl radical upon donating a hydrogen atom that could quench active free radicals and stop the propagation of lipid peroxidation [9,10]. The number and position of aromatic hydroxyl groups were found to have strong impact on the activity of phenolic antioxidants [11-13]. The presence of *o*-hydroxyl group was reported to lower the O-H bond dissociation energy and hence increase the hydrogen atom donation ability [13-16] and considered the most important structural feature of the high activity [11,17]. Electron donating groups, especially alkyl and methoxy groups were reported to increase the electron density of the phenoxyl radicals leading to enhancement of the radical scavenging and antioxidant activity [18]. The high activity of α-tocopherol was attributed to the *p*-alkoxy group and the methyl groups on the aromatic ring [19].

Fifty one structurally variant phenolic and anilinic compounds were examined as radical scavengers against DPPH, hydroxyl and peroxyl radicals, and as antioxidants in sunflower oil and liver homogenate systems. Kinetics *vs* thermodynamics of scavenging hydroxyl radical was examined.

## Experimental

### Chemicals

All chemicals were obtained as reagent grade from Aldrich, Sigma or Fluka chemical companies and used without further purification.

### DPPH radical scavenging activity

The 2, 2-diphenyl-1-picrylhydrazyl (DPPH) radical scavenging ability of the examined compounds was measured according to Brand-Williams, Cuvelier, & Berset [20]. The examined compound (25 μL, 5 mM) or 25 μL methanol (as a control) with 2.5 ml 0.004% DPPH in methanol (0.1 mM), were mixed. The solution was incubated for 20 min at room temperature before reading the absorbance (A) at 517 nm against methanol as blank. The inhibitory percentage of DPPH was calculated according to the following equation:

$$\begin{array}{l} \%\;\mathrm{DPPH}\;\mathrm{radical}\;\mathrm{ascavinging}\;\mathrm{activity}\\ \;=100-\left(\frac{A517\;\left(\mathrm{Exp}\right)}{A517\;\left(\mathrm{control}\right)}\times \;100\right) \end{array}$$

### % Hydroxyl radical scavenging activity

Hydroxyl radical scavenging activity of the examined compounds was measured based on the method of Halliwell, Gutteridge, & Arouma [21], with a slight modification according to Jiang, Bank, & Scholes [22]. Briefly, 200 μL deoxyribose solution (2.8 mM), 200 μL H_2_O_2_ (1.4 mM) and 200 μL of the examined compound (5 mM) or oxygen free water (control), were placed in a test tube. Fenton reaction was initiated by the addition of 200 μL EDTA (100 μM), 200 μL FeCl_2_ solution (20 μM in 1 mM HCl); all used solutions were oxygen free. The total volume of the reaction mixture (1 ml) was mixed and incubated for 10 second at room temperature. The reaction was stopped by the addition of 1 ml 10% trichloroacetic acid (TCA), then 1 ml 1% thiobarbituric acid (TBA) solution in 50 mMNaOH containing 0.02% butylatedhydroxyanisole (BHA) was added. The mixture was heated at 80°C for 15 minutes then cooled and the absorbance (A) was measured at 532 nm. The hydroxyl radical scavenging activity was calculated according to the following equation:

$$\begin{array}{l} \%\;\mathrm{OH}\;\mathrm{radical}\;\mathrm{scavinging}\;\mathrm{activity}\\ \;=100-\left(\frac{A532\;\left(\mathrm{Exp}\right)}{A532\;\left(\mathrm{control}\right)}\times \;100\right) \end{array}$$

### Rate constant of scavenging hydroxyl radical

The rate constant (ks) of hydroxyl radical scavenging reaction was measured by using the previous method of the deoxyribose model [21]. The same procedure was adopted by using various concentrations of the examined compounds (0–1 mM final concentration), and then the following equation was applied using a linear regression analysis by plotting 1/A *vs* [S].

Where: A is the absorbance in the presence of the examined compound, Aoisthe absorbance in the absence of the examined compound, k_DR_is the second order rate constant of the reaction of deoxyribose with hydroxyl radical (3.1 × 10^6^M^-1^Sec^-1^), [S] is the molar concentration of the scavenger, [DR] is the molar concentration of deoxyribose (0.56 mM), and ks is the second order rate constant (mM^-1^Sec^-1^) of the reaction of a compound with the hydroxyl radical.

### Peroxyl radical scavenging activity

Scavenge peroxyl free radicals was estimated by ORAC method described by Cao & Prior [23] and modified by Gerhäuser et al [24]. AAPH, 2,2-azobis (2-amidinopropane) dihydrochloride, was used as peroxyl radical generator while β-phycoerythrin (β-PE) was used as a redox-sensitive fluorescent indicator protein. Fluorescence was measured at 37°C for 100 min until completion using Microplate reader FluoStarOptimum with excitation at 540 nm and emission at 565 nm. Trolox was used as standard where one ORAC unit is equal to the net protection of β-PE produced by 1 μM trolox.

### Antioxidant activity in sunflower oil

An antioxidant activity assay was carried out in sunflower oil as described by Osawa & Namiki [25], with slight modification. The examined compound (0.2 ml, 5 mM in Methanol) or 0.2 ml methanol (control) was added to a solution of sunflower oil (0.2 ml oil, 10 ml 99.8% ethanol and 10 ml 0.2 M phosphate buffer pH 7.0). The total volume was adjusted to 25 ml with distilled water. The reaction mixture was incubated at 30°C for 24 hrs, then the degree of oxidation was measured according to the thiocyanate method by sequentially adding ethanol (10 ml, 75%), ammonium thiocyanate (0.2 ml, 30%), sample solution (0.2 ml), and ferrous chloride (0.2 ml, 20 mM in 3.5% HCl). After the mixture was stirred for 3 minutes, the peroxide value was determined by reading the absorbance (A) at 500 nm. The percentage inhibition of oil acid peroxidation was calculated according to the following formula:

$$\%\;\mathrm{Antioxidant}\;\mathrm{activity}=100-\left(\frac{A500\;\left(\mathrm{Exp}\right)}{A500\;\left(\mathrm{control}\right)}\times \;100\right)$$

### Antioxidant activity in rat liver

Albino rabbit liver was used. Liver tissue (4 g) was sliced and homogenized in 22.5 ml KCl-Tris HCL buffer (150 mM, pH 7.2) and centrifuged at 5000 × g for 10 minutes to give supernatant of liver homogenate. The effect of anti-FeCl_2_-ascorbic acid stimulated lipid peroxidation was determined by the method of Yoden, Lio, & Tabata [26]. The liver homogenate (0.4 ml), 0.1 ml Tris–HCl buffer (pH 7.2) and 0.2 ml tested compound (5 mM in methanol) or 0.2 ml methanol (control) were mixed and incubated for 1 h at 37°C. After incubation, 0.5 ml 0.1 N HCl, 0.5 ml 0.9% sodium dodecyl sulfate (SDS), and 0.5 ml H_2_O were added and shaken with the incubation solution. TBA (2 ml, 0.5%) was added then the mixture was heated for 30 minutes in a boiling water bath. After cooling, 5 ml *n*-butanol was added; the mixture was then shaken vigorously. The *n*-butanol layer was separated by centrifugation at 1000 × g. The absorbance (A) of the sample was read at 532 nm against a blank, which contained all reagents except antioxidant.

### Theoretical calculations and statistical analysis

Heat of formation was calculated by MOPAC calculations using PM3 method in CambridgeSoft package (2000) after energy minimization. The electrophilic Brown parameter (σ^+^_p_) of *para* substituents and Hammett parameter (σm) of *meta* substituents were obtained from the literature [27], while σ^+^_o_ for *ortho* substituents was calculated from the formula σ^+^_o_ = 0.66 σ^+^_p_[28].

Regression analyses were performed using SPSS software version 16. Correlations were assessed by the correlation coefficient (R^2^), standard error of the estimate (SE), the number of data point (N), the least significant difference (*p*), and the 95%-confidence intervals (in parentheses) for each regression coefficient.

## Results and discussion

**Figure 1:**
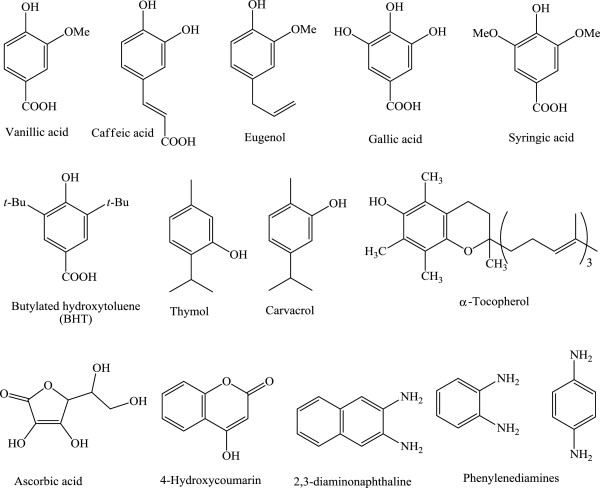
Structures of some of the examined phenolic, enolic and anilinic compounds.

Antioxidant and radical scavenging activities against DPPH, hydroxyl and peroxyl radicals of a wide range of natural (e.g. α-tocopherol, eugenol, thymol, carvacrol, caffeic acid, vanillic acid syringic acid and gallic acid) and synthetic phenolic compounds were examined; the results are presented in Table 1. The structural diversity of the used phenolic compounds (Figure 1) includes monophenols with substituents frequently present in natural phenols e.g. alkyl, alkoxy, ester and carboxyl groups, besides many other electron donating and withdrawing groups; in addition to polyphenols with 1–3 hydroxyl groups and aminophenols. Some common groups e.g. alkyl, carboxyl, amino and second OH groups were incorporated in *ortho*, *meta* and *para* positions. Substituted anilines and enols (e.g. ascorbic and 4-hydroxycoumarin) as structurally and chemically related to phenols were also included.

**Table 1:** Radical scavenging and antioxidant activities of phenols, enols and anilines

**Entry**	**Compound**	**% DPPH inh.**	**Peroxyl inh.**	**% OH inh.**	**ks**^**a**^	**% AO**^**b **^**activity**	**% AO**^**c **^**activity**
1	Phenol	2.3	0.0	0.4	0.83	0.0	0.0
2	Catechol	98.0	1.8	70.0	402.59	54.0	49.0
3	Resorcinol	2.5	0.8	5.1	9.40	4.0	5.0
4	Hydroquinone	97.0	2.1	67.0	360.50	54.0	47.0
5	2- Aminophenol	92.0	1.49	63.1	307.42	46.0	40.0
6	3-Aminophenol	20.2	0.7	7.3	13.82	7.0	2.0
7	4- Aminophenol	97.0	1.4	65.0	333.41	44.0	40.0
8	3- Nitrophenol	1.3	0.89	2.9	5.36	5.0	4.0
9	4- Nitrophenol	1.1	0.0	5.6	10.51	5.0	6.0
10	3-Chlorophenol	3.1	1.1				
11	4- Chlorophenol	3.6	0.81	1.7	3.04	0.0	0.0
12	4-Hydroxybenzaldehyde	1.1	0.3	4.6	8.29	2.0	3.0
13	2-Hydroxyacetophenone	0.0					
14	Phenol-4-sulfonic acid	0.3	0.9	3.4	6.08	1.0	1.0
15	Salicylic acid	1.3	0.9	8.9	17.14	1.0	2.0
16	3-Hydroxybenzoic acid	2.5	0.0	6.9	12.72	4.0	3.0
17	4-Hydroxybenzoic acid	1.8	0.56	2.5	4.48	3.0	1.0
18	Ethyl salicylate	1.2	0.81				
19	Methyl salicylate	1.6	0.77				
20	Ethyl 4-hydroxybenzoate	1.9	0.47				
21	3,5-Dinitrosalicylic acid	0.8	0.0	4.3	7.19	0.0	1.0
22	3,4-Dihydroxybenzoic	89.6	2.41	35.4	93.44	57.0	51.0
23	2,5-Dihydroxybenzoic	95.0	2.62	49.0	88.47	63.0	60.0
24	Ethyl 2,5-dihydroxybenzoate	96.1	2.5				
25	*o*-cresol	12.2	0.62				
26	*p*- Cresol	15.5	0.52				
27	Thymol	35.0					
28	Carvacrol	33.9					
29	Butylatedhydroxytoluene (BHT)	96.0	2.98	95.0	361.61	66.0	59.0
30	α-Tocopherol	96.2		68.8	373.77	47.0	45.0
31	Eugenol	98.1		97.0	389.81	63.0	60.0
32	Guaiacol(*o*-methoxyphenol)	28.3					
33	Vanillic acid	25.1	0.8	2.7	5.03	2.1	2.3
34	Syringic acid	90.4	2.9	62.2	283.09	58.0	52.0
35	Gallic acid	92.0	3.09	65.0	331.20	61.0	59.0
36	Caffeic acid	91.2	2.78	53.5	196.29	62.0	57.0
37	Ascorbic acid	99.1	2.94	81.0	361.61	46.0	49.0
38	4- hydroxycoumarin	1.5		2.4	3.59	0.0	0.0
39	1-Naphthol	35.0	1.23				
40	Aniline	8.7		8.6	16.59	2.0	6.0
41	*p*-Tuluidine	18.4					
42	*p*-Anisidine	31.1		4.9	8.85	6.0	3.0
43	*p*-Phenylenediamine	90.1		66.0	347.78	43.0	49.0
44	*o*-Phenylenediamine	5.1		2.8	5.032	3.0	1.0
45	2,3-Diaminophethaline	2.9		5.5	9.95	5.0	4.0
46	Anthranillic acid	5.7					
47	*p*-Aminobenzoic acid	4.6					
48	*m*-Bromoaniline	0.0					
49	*p*-Chloroaniline	0.0					
50	*o*-Nitroaniline	0.0					
51	*p*-Nitroaniline	0.0					

^a^ Bimolecular rate constant, ks × 10^-7^(M^-1^s^-1^).

^b^ % Antioxidant activity in sunflower system.

^c^ % Antioxidant activity in liver homogenate system.

### Structure-activity relationships (SAR)

DPPH has frequently been used as a reactive hydrogen acceptor for the determination of radical scavenging activity of various natural and synthetic compounds [29]. Results listed in Table 1 indicate that the examined compounds (51 compounds) expressed anti DPPH radical activity ranged from 0 to 99.1%, 15 of them (entries 2, 4, 5, 7, 22–24, 29–31, 34–37, 43) exhibited high scavenging activity (> 89.6). The fifteen highly active compounds possessed some special structural features that can be depicted in the following structure-activity relationships: (1) all polyphenols (7 compounds) with a second hydroxyl group in the *ortho* or *para* positions e.g. catechol and hydroquinone showed high activity (98 and 97% respectively); however, the *meta* isomer (resorcinol) showed very low activity (2.5%). This result can be explained by the strong electron donation ability of the hydroxyl group in *ortho* and *para* positions (σ_p_^+^ = − 0.92, σ_o_^+^ = − 0.61) while the hydroxyl in *meta* position is e.w.g. (σm = 0.12). It is well established that electron donating groups stabilize the resulted phenoxyl radicals through inductive (as in alkyl substituents) or resonance (as in OMe or NH_2_ substituents) effect; thus lower the O-H bond energy and enhance the radical scavenging activity. In contrary, electron withdrawing groups stabilize more the phenols and destabilize the resulted radicals [30-32]. In addition, a hydrogen bonding can be formed between the phenoxyl unpaired electron and the adjacent hydroxyl group in catechols that stabilizes the radicals formed more than it does for the parent diols [33-35]. It is also known that catechol and hydroquinone undergo two hydrogen-atom transfer process to give the stable *o*- and *p*-quinones respectively [10]. Ascorbic acid is well known good antioxidant [36] and exhibited high activity reached 99.1%. It could also undergo the two hydrogen-atom transfer process to give the dehyroascorbic acid [37]. (2) Similarly, *o-* and *p*-aminophenols showed high activity (92 and 97% respectively) because of the strong electron donating effect of the amino group in these positions (σ_p_^+^ = − 1.30, σ_o_^+^ = − 0.86) and the possible formation of 2- and 4-iminoketones respectively. However, *m-*aminophenol gave moderate activity (20.2%) compared to resorcinol (2.5%), since the amino group is still e.d.g. in the *m-*position (σm = − 0.16) while the hydroxyl group is e.w.g. (σm = 0.12) in the same position. (3) Most other monophenols with various substituents e.g. carboxyl (entries 15–17), ester (entries 18–20), CHO (entry 12), CH_3−_CO (entry 13), SO_3_H (entry 14), NO_2_ (entries 8–9) and Cl (entries 10–11) gave low activity (< 3.6%). However, the presence of one alkyl group as in *o*- and *p*-cresol raised the activity to 12.2 and 15.5% respectively compared to that of phenol (2.3%), while two alkyl groups as in thymol and carvacrol (in *o-* and *m*-positions) increased the activity to 35.0 and 33.9% respectively; these results indicate that alkyl groups in any position (*o*, *m* or *p*) stabilize the phenoxyl radicals through inductive effect and thus enhance the antradical activity. The activity of thymol and carvacrol was previously reported [38]. Although three alkyl groups in BHT gave high activity (96.0%), this activity is exceptionally high where BHT showed special antiradical mechanism [39]. The presence of methoxy group alone (guaiacol) or with carboxyl group (vanillic acid) gave moderate activity (28.3 and 25.1% respectively) while two methoxy groups in syringic acid gave high activity (90.4%). This result confirmed the impact of alkyl and alkoxy groups as good e.d.g. in increasing the electron density and stabilizing of the phenoxyl radicals. However, it should be noted that methoxy group, in contrary to alkyl groups, has to be in *o-* or *p-*position to act as good e.d.g. as indicated by Brown parameter (σ_p_^+^ = − 0.78, σ_o_^+^ = − 0.51). On the other hand, the *m*-methoxy group works normally as e.w.g. as indicated by sigma parameter (σm = 0.12) but the strong electron withdrawing activity of an oxygen phenoxyl radical causes the *m*-methoxy group to become weak e.d.g., σ_m_^+^ = −0.14 [40]. These results suggest that both alkyl and alkoxy groups, in the proper positions, seem to enhance the activity in additive way which could be applied and examined in a QSAR study (Ali & Ali, personal communications). (4) Anilines gave the same trends as phenols; some of the preliminary aniline antioxidant activity results were previously reported [41]. *p*-Phenylenediamine, as might be expected, gave high activity (90.1%) as a result of electron donating effect of the amino group and the possible two hydrogen-atom transfer process leading to the formation of 1,4-diimine; however, *o*-phenylenediamine gave low activity (5.1%) which could be attributed to the strain energy manifested by the relatively high heat of formation of the 1,2-diimine (ΔH_F_ = 119.47 Kcal/mole) formed from the *o*-isomer compared to that of 1,4-diimine (ΔH_F_ = 73.41 Kcal/mole) resulted from the *p*-isomer as deduced by MOPAC calculations. This result is confirmed by the low activity expressed by the 2,3-diaminonaphthaline (2.9%). As in substituted phenols, aniline has low activity (8.7%) while the *p*-methyl group in *p*-toluidine raised the activity to 18.4% and *p*-methoxy in *p*-anisidine has even higher effect (31.1%); other substituents (carboxyl, bromo, chloro or nitro substituents) showed little negative effect.

### Scavenging hydroxyl and peroxyl radicals

**Figure 2:**
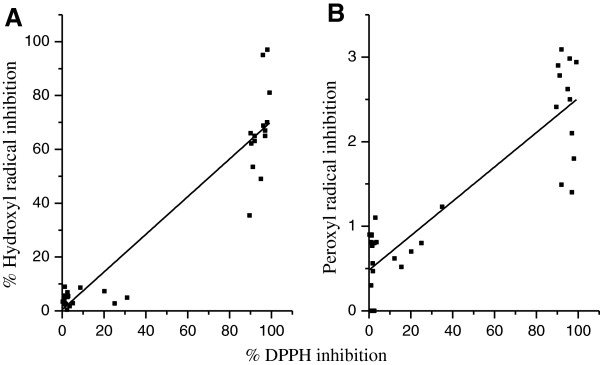
Correlations between % DPPH inhibition and each of % of hydroxyl (A) and peroxyl (B) radical inhibition.

To examine the validity of using the simple DPPH test as indicator for the activity towards other radicals, scavenging the OH radical, one of the most reactive radicals presents in living cells [42,43], and peroxyl radical, usually formed naturally upon lipid peroxidation [15], were determined; the results are listed in Table 1. The trends of scavenging both hydroxyl and peroxyl radicals are much similar to that of DPPH radical as presented by eqs. 1 and 2 respectively and plotted in Figure 2.

(1) $$\begin{array}{l} \%\;\mathrm{OH}\;\mathrm{inhibition}=0.46\;\left(\pm 2.77\right)\\ \;+0.70\;\left(\pm 0.04\right)\;\%\;\mathrm{DPPH}\;\mathrm{inhibition}\\ N=32,R^{2}=0.894,\mathrm{SE}=11.01,p<0.001 \end{array}$$

(2) $$\begin{array}{l} \mathrm{Peroxyl}\;\mathrm{radical}\;\mathrm{inhibition}=0.48\;\left(\pm 0.11\right)\\ \;+0.02\;\left(\pm 0.00\right)\;\%\;\mathrm{DPPH}\;\mathrm{inhibition}\\ N=32,R^{2}=0.793,\mathrm{SE}=0.46,p<0.001 \end{array}$$

### Thermodynamic vs kinetic of OH radical scavenging activity

**Scheme 1:**
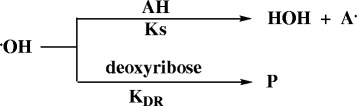
**Possible reactions of hydroxyl radical in the reaction mixture.** Reaction rate = ks [OH] [AH] + k_DR_[OH] [DR].

Although the % hydroxyl radical scavenging activity determines the ability of antioxidant to scavenge the hydroxyl radicals, it does not give direct measure of the intrinsic reactivity of these antioxidants. Good antioxidants should have high scavenging activity (thermodynamic property) and relatively high reaction rate (kinetic property); therefore, the second order rate constant (ks) of the H-atom transfer from antioxidant to the hydroxyl radical was determined in the deoxyribose assay. In this assay the hydroxyl radicals may react with either the antioxidant (AH) or the deoxyribose (DR) in parallel fashion mechanism (Scheme 1).

**Figure 3:**
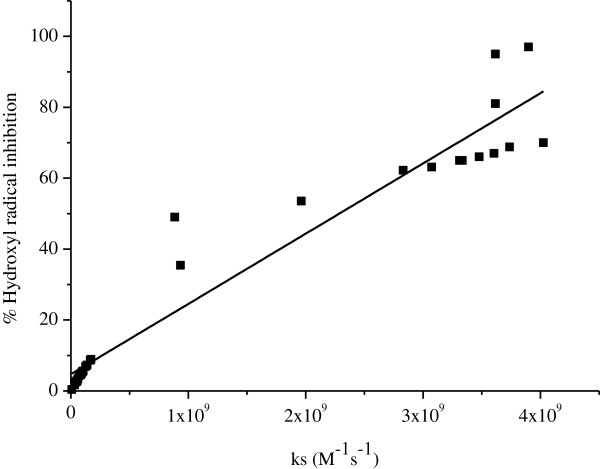
Correlation between % OH radical inhibition and the rate constant (ks).

The rate constants (ks) listed in Table 1 show, as expected, a high reactivity of the hydroxyl radical towards most compounds even those with low scavenging activity, expressed by high rate constant that ranged from 8.29 × 10^6^ (phenol) - 4.03 × 10^9^ (catechol) M^-1^s^-1^. In addition, compounds with the highest DPPH and OH radical scavenging activities showed also the highest rate constant (8.85 × 10^8^ – 4.03 × 10^9^ M^-1^ s^-1^). The strong correlation between % of OH radical inhibition and rate constant is presented by eq. 3 and Figure 3.

(3) $$\begin{array}{l} \%\;\mathrm{OH}\;\mathrm{radical}\;\mathrm{inhibition}=4.72\;\left(\pm 1.98\right)\\ \;+1.98\times 10^{-8}\left(\pm 0.00\right)\;\mathrm{ks}\\ N=32,R^{2}=0.937,\mathrm{SE}=8.52,p<0.001 \end{array}$$

### Antioxidant activity

**Figure 4:**
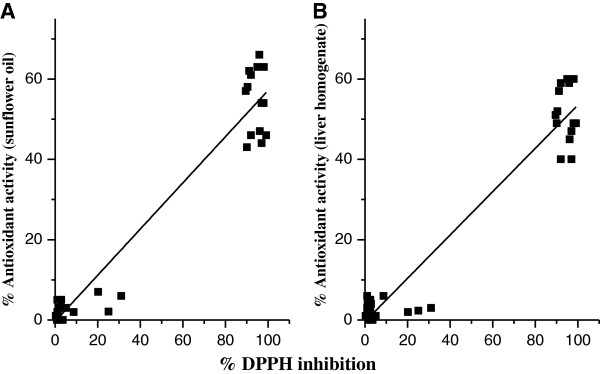
Correlations between % DPPH inhibition and the antioxidant activity against lipid peroxidation in each of sunflower oil (A) and liver homogenate (B).

Antioxidant activity against lipid peroxidation in two systems, sunflower oil and liver homogenate, was determined (Table 1). The strong radical scavengers showed also good antioxidant activity which implies similar structural requirements and the dependence of antioxidant activities on radical scavenging activities. The strong correlations between antioxidant activities in both systems and DPPH inhibition is presented by eqs. 4 and 5 respectively, and Figure 4.

(4) $$\begin{array}{l} \%\;\mathrm{antioxidant}\;\mathrm{activity}\;\left(\mathrm{sunflower}\;\mathrm{oil}\right)=-0.34\;\left(\pm 1.66\right)\\ \;+0.58\;\left(\pm 0.03\right)\;\%\;\mathrm{DPPH}\;\mathrm{inhibition}\\ N=32,R^{2}=0.941,\mathrm{SE}=6.61,p<0.001 \end{array}$$

(5) $$\begin{array}{l} \%\;\mathrm{antioxidant}\;\mathrm{activity}\;\left(\mathrm{liver}\;\mathrm{homogenate}\right)=-0.39\;\left(\pm 1.57\right)\\ \;+0.54\;\left(\pm 0.03\right)\;\%\;\mathrm{DPPH}\;\mathrm{inhibition}\\ N=32,R^{2}=0.940,\mathrm{SE}=6.25,p<0.001 \end{array}$$

Therefore, presence of good e.d.g. with the ability to form stable quinone-like product or the presence of at least three alkyl or two alkoxy groups is still required for phenols or anilines to possess good antioxidant activity in oils or living cells.

## Conclusions

These results imply that the structural features and factors required for good anti DPPH activity are also required for both anti OH and peroxyl radical activities and antioxidant activities in various systems. The factors are either the presence of *o*- or *p*-hydroxyl or amino groups that could form quinone-like product, or the presence of a number of alkoxy (in *o* or *p*-position) and/or alkyl groups (in *o*, *m* or *p*-position) to stabilize the resulted phenoxyl radical. Kinetic measurements showed that strong antioxidants with high activity have also high reaction rates indicating that factors stabilizing the phenoxyl radicals lower also the activation energy.

## Competing interests

The authors declare that they have no competing interests.

## Authors’ contributions

HMA designed the experiments, performed the SAR analysis and wrote the paper. AAS and WGS specified the research point and aims. HASE and HAS contributed to the experimental design and techniques, OAAB and AMS ran the experiments. All authors read and approved the final manuscript.

## References

[B1] Balasundram N Sundram K Samman S Phenolic compounds in plants and agro industrial by-products: Antioxidant activity, occurrence, and potential uses Food Chem 2006 99 191 203 10.1016/j.foodchem.2005.07.042

[B2] Khan HRA Khan MR Sahreen S Ahmed M Evaluation of phenolic contents and antioxidant activity of various solvent extracts of Sonchusasper L Chemistry Central Journal 2012 6 12 18 10.1186/1752-153X-6-12 22305477 PMC3292812

[B3] Božin B Mimica-Dukić N Samojlik I Anačkov G Igić R Phenolics as antioxidants in garlic (Allium sativum L., Alliaceae) Food Chem 2008 111 925 929 10.1016/j.foodchem.2008.04.071

[B4] Orèiæ DZ Mimica-Dukiæ NM Franciškoviæ MM Petroviæ SS Jovin EÐ Antioxidant activity relationship of phenolic compounds in Hypericumperforatum L Chemistry Central Journal 2011 5 34 41 10.1186/1752-153X-5-34 21702979 PMC3132159

[B5] Gizdavic-Nikolaidis M Travas-Sejdic J Kilmartin PA Bowmaker GA Cooney RP Evaluation of antioxidant activity of aniline and polyaniline Curr Appl Phys 2004 4 343 346 10.1016/j.cap.2003.11.044

[B6] Ismail MN Ibrahim MS Abd El-Ghaffar MA Polyaniline as an antioxidant and antirad in SBR vulcanizates Polym Degrad Stab 1998 62 337 341 10.1016/S0141-3910(98)00016-0

[B7] Ali HM El-Qurashi MAM Synthesis and application of some dianilinosilanes, bis (trimethylsilyl) phenylenediamines and dialkylbenzo-1,3,2-diazasilolines as antioxidants Phosphorus, Sulfur and Silicon 1998 134/135 521 529

[B8] El-Qurashi MAM Ali HM Anilinosilanes as thermo-oxidation stabilizers of commercial lubricating base oils Thermochimica Acta 1997 293 185 190 10.1016/S0040-6031(96)03092-4

[B9] Cheng Z Ren J Li Y Chang W Chen Z Establishment of a quantitative structure-activity relationship model for evaluating and predicting the protective potentials of phenolic antioxidants on lipid peroxidation J Pharmaceutical Sci 2003 92 475 484 10.1002/jps.10301 12587109

[B10] Foti M Ruberto G Kinetic solvent effects on phenolic antioxidants determined by spectroscopic measurements J Agric Food Chem 2001 49 342 348 10.1021/jf0006527 11170597

[B11] Cai Y-Z Sun M Xing J Luo Q Corke H Structure-radical scavenging activity relationships of phenolic compounds from traditional Chinese medicinal plants Life Science 2006 78 2872 2888 10.1016/j.lfs.2005.11.004 16325868

[B12] Lien EJ Ren S Bui H-H Wang R Quantitative structure-activity relationship analysis of phenolic antioxidants Free Radic Biol Med 1998 26 285 294 9895218 10.1016/s0891-5849(98)00190-7

[B13] Tyrakowska B Soffers AEMF Szymusiak H Boeren S Boersma MG Lemanska K Vervoort J Rietjens IMCM TEAC antioxidant activity of 4-hydroxybenzoates Free Radic Biol Med 1999 27 1427 1436 10.1016/S0891-5849(99)00192-6 10641737

[B14] De Pinedo AT Peňalver P Morales JC Synthesis and evaluation of new phenolic-based antioxidants: structure-activity relationship Food Chem 2007 103 55 61 10.1016/j.foodchem.2006.07.026

[B15] Amorati R Pedulli GF Cabrini L Zambonin L Landi L Solvent and pH effects on the antioxidant activity of caffeic and other phenolic acids J Agric Food Chem 2006 54 2932 2937 10.1021/jf053159+ 16608211

[B16] Lucarini M Pedulli GF Bond dissociation enthalpy of α-tocopherol and other phenolic antioxidants J Org Chem 1994 59 5063 5070 10.1021/jo00096a061

[B17] Ordoudi SA Tsimidou MZ Vafiadis AP Bakalbassis EG Structure-DPPH scavenging activity relationships; parallel study of catechol and guaiacol acid derivatives J Agric Food Chem 2006 54 5763 5768 10.1021/jf060132x 16881675

[B18] Kajiyama T Ohkatsu Y Effect of para-substituts of phenolic antioxidants Polym Degrad Stab 2001 71 445 452 10.1016/S0141-3910(00)00196-8

[B19] Breese KD Lamѐ the J-F DeAmitt C Improving synthetic hindered phenol antioxidants: learning from vitamin E Polym Degrad Stab 2000 70 89 96 10.1016/S0141-3910(00)00094-X

[B20] Brand-Williams W Cuvelier ME Berset C Use of a free radical method to evaluate antioxidant activity Food Sci Technol 1995 28 25 30

[B21] Halliwell B Gutteridge JMC Arouma OI The deoxyribose method: a simple “test-tube” assay for determination of rate constants for reactions of hydroxyl radicals Anal Biochem 1987 165 215 219 10.1016/0003-2697(87)90222-3 3120621

[B22] Jiang J Bank JF Scholes CP Structure and function of lipid soluble vitamins J Am Chem Soc 1993 115 4742 4746 10.1021/ja00064a038

[B23] Cao G Prior RL The measurement of oxygen radical absorbance capacity in biological samples Meth Enzymol 1999 299 50 62 9916196 10.1016/s0076-6879(99)99008-0

[B24] Gerhäuser C Klimo K Heiss E Neumann I Gamal-Eldeen A Knauft J Liu GY Sitthimonchai S Frank N Mechanism-based in vitro screening of potential cancer chemopreventive agents Mutat Res 2003 523–524 163 172 12628514 10.1016/s0027-5107(02)00332-9

[B25] Osawa T Namiki M A Novel type of antioxidant isolated from leaf wax of Eucalyptus leaves J Agric Food Chem 1981 45 735 739

[B26] Yoden K Lio T Tabata T Measurment of thiobarbituric acid value in tissue homogenate solutized with sodium dodecyl sulphate Yakugaku Zasshi 1980 100 553 559 7452447 10.1248/yakushi1947.100.5_553

[B27] Hansch C Leo A Exploring QSAR Fundamental And Applications in Chemistry And Biology 1995 Washington, DC: American Chemical Society

[B28] Jonsson M Lind J Eriksen TE Merenyi G O–H bond strengths and one-electron reduction potentials of multisubstituted phenols and phenoxy radicals. Predictions using free energy relationships J Chem Soc, Perkin Trans II 1993 1567 1568

[B29] Molyneux P The use of the stable free radical diphenylpicrylhydrazyl (DPPH) for estimating antioxidant activity Songklanakarin J Sci Technol 2004 26 211 219

[B30] Foti MC Daquino C Mackie ID DiLabio GA Ingold KU Reaction of phenols with the 2,2-diphenyl-1-picrylhydrazyl radical. Kinetics and DFT calculations applied to determine ArO-H bond dissociation enthalpies and reaction mechanism J Org Chem 2008 73 9270 9282 10.1021/jo8016555 18991378

[B31] Foti MC Antioxidant properties of phenols J Pharm Pharmacol 2007 59 1673 1685 10.1211/jpp.59.12.0010 18053330

[B32] Lucarini M Pedulli GF Free radical intermediates in the inhibition of the autoxidation reaction Chem Soc Rev 2010 39 2106 2119 10.1039/b901838g 20514720

[B33] Foti MC Johnson ER Vinqvist MR Wright JS Barclay LRC Ingold KU Naphthalene diols: a new class of antioxidants intramolecular hydrogen bonding in catechols, naphthalene diols, and their aryloxyl radicals J Org Chem 2002 67 5190 5196 10.1021/jo020184v 12126405

[B34] Foti MC Barclay LRC Ingold KU The role of hydrogen bonding on the H-atom-donating abilities of catechols and naphthalene diols and on a previously overlooked aspect of their infrared spectra J Am Chem Soc 2002 124 12881 12888 10.1021/ja020757l 12392436

[B35] Amorati R Valgimigli L Modulation of the antioxidant activity of phenols by non-covalent interactions Org Biomol Chem 2012 10 4147 4158 10.1039/c2ob25174d 22505046

[B36] Amorati R Pedulli GF Valgimigli L Kinetic and thermodynamic aspects of the chain-breaking antioxidant activity of ascorbic acid derivatives in non-aqueous media Org Biomol Chem 2011 9 3792 3800 10.1039/c1ob05334e 21479296

[B37] Brunhuber NMW Mort JL Christoffersen RE Reich NO Steady-state kinetic mechanism of recombinant avocado ACC oxidase: initial velocity and inhibitor studies Biochem 2000 39 10730 10738 10.1021/bi0000162 10978157

[B38] Yanishlieva NV Marinova EM Gordon MH Raneva VG Antioxidant activity and mechanism of action of thymol and carvacrol in two lipid systems Food Chem 1999 64 59 66 10.1016/S0308-8146(98)00086-7

[B39] Bondet V Brand-Williams W Berset C Kinetics and mechanisms of antioxidant activity using the DPPH free radical method Food Sci Technol 1997 30 609 615

[B40] Foti MC Daquino C DiLabio GA Ingold KU A meta effect in nonphotochemical processes: the homolytic chemistry of m-methoxyphenol. J Org Chem 2008 73 2408 2411 b) *ibid*. *J Org Chem* 2008, **73:**7440–7440 10.1021/jo702520r 18294001

[B41] Abo Shady A Ali HM Sharaf EHA Abdel-Barry OA DPPH and hydroxyl radical scavenging and antioxidant activities of anilines and related compounds Bull Biol Chem Environ Sci 2007 2 257 266

[B42] Cheng Z Ren J Li Y Chang W Chen Z Study on the multiple mechanisms underlying the reaction between hydroxyl radical and phenolic compounds by quantitative structure and activity relationship Bioorg Med Chem 2002 10 4067 4073 10.1016/S0968-0896(02)00267-5 12413860

[B43] Korycka-Dahi MB Richardson T Activated oxygen species and oxidation of food constituents Crit Rev Food Sci Nutr 1978 10 209 241 10.1080/10408397809527250 215383

